# Implementation of a Pediatric Oncology Precision Medicine Clinic to Personalize Approaches for Diagnosing and Treating Solid Tumors

**DOI:** 10.32604/or.2025.065547

**Published:** 2025-07-18

**Authors:** Madeline Keane, Natalia Wojciechowska, Lindsay Zumwalt, Emilie Sandfeld, Alejandra Dominguez, Jason Wang, Anish Ray

**Affiliations:** 1 Anne Burnett Marion School of Medicine at Texas Christian University, Fort Worth, TX 76104, USA; 2 Department of Pediatric Oncology, Cook Children’s Medical Center, Fort Worth, TX 76104, USA; 3 College of Medicine, Texas A&M University, Dallas, TX 75246, USA

**Keywords:** Precision medicine, next generation sequencing (NGS), targeted therapies, pediatric oncology

## Abstract

**Background:**

Precision medicine is an emerging approach for treating pediatric cancer due to its ability to target tumor-specific genetic drivers rather than provide broad and aggressive treatments. The study aimed to outline the establishment and impact of a Precision Medicine Clinic (PMC) in the setting of pediatric oncology, with the objective of offering targeted treatment options within the institution and creating a scalable model for adoption by other healthcare systems to achieve a wider impact.

**Methods:**

Recognizing this need for an individualized approach to treating patients, Cook Children’s Medical Center (CCMC) established a multidisciplinary molecular tumor board in 2019, followed by the launch of an official PMC in 2021. Before this, there was no dedicated place to discuss and evaluate genetic sequencing results.

**Results:**

In 2022 and 2023, the PMC discussed 69 patients with a wide variety of oncologic diagnoses. Through the clinic’s efforts, 133 genetic variants across 101 genes have been identified, spanning oncogenic pathways related to cell cycling, DNA processing, and cell signaling. Of the sequenced patients, four have received targeted therapy according to recommendations from the PMC.

**Conclusion:**

While the PMC continues to evaluate patients and their long-term outcomes, the continually growing PMC at CCMC represents the beginning of the advancement of treating pediatric oncology patients through the interpretation of genetic sequencing results, making actionable targeted treatment recommendations, and continuing to follow the patient’s course of care over time. This additionally provides a framework for starting a PMC that can be adapted for specific clinical needs and implemented broadly.

## 1 Introduction

While survival rates for childhood cancers have significantly improved over the last few decades, the increasing number of survivors has also led us to understand the long-term physical and psychological health challenges resulting from early-life exposure to aggressive treatments [1,2]. To mitigate many of the adversities faced, there has been substantial progress in uncovering tumor-specific genetic drivers to design therapies that selectively eliminate malignant cells while preserving healthy tissue. This strategy, often referred to as precision or targeted therapy [3,4], continues to gain traction as a critical component of pediatric cancer care. Emerging systems-based and network biology approaches are further refining how molecular targets are identified and integrated into therapeutic development in pediatric oncology [5,6]. By leveraging genomic sequencing and tumor markers, there is an enhancement in diagnostic precision, targeted therapies can be applied such that effectiveness is optimized, and there can be a reduction in the long-term adverse effects commonly associated with conventional chemotherapy and radiation [7,8].

Historically, the standard treatment modalities for pediatric cancers have focused on combinations of surgery, chemotherapy, and radiation, all of which remain the backbone of care for most malignancies despite associated late effects [9]. Advancements such as immunotherapy and targeted therapies are increasingly supplementing conventional regimens, aiming to improve outcomes while reducing long-term toxicities [10].

With the development of the discipline of precision medicine, a drastic rise in research, use, and funding of genomic testing, as part of the National Cancer Moonshot Initiative, has helped to increase the acceptance of precision medicine as a specialized field, particularly in the realm of pediatric oncology [11]. Integrating next-generation sequencing into pediatric oncology care allows for diagnostic refinement and can identify actionable mutations that can alter therapeutic decisions in a substantial portion of patients [12,13]. There has been a fortuitous convergence of opportunity, technology, and shifting attitudes leading to the relative boom in precision medicine [14]. First, biomedical research has witnessed the generation of a large amount of data, together with information technology becoming more user-friendly and widely available. Electronic health records have made it possible for massive amounts of data about patients to be compiled and compared, allowing for correlations to be found among large groups. Finally, increased receptivity toward molecular data and changes in public attitudes regarding the privacy of healthcare information have led to increased acceptance of precision medicine’s aims [15].

Meanwhile, targeted cancer therapies have been shown to have astounding success. Trastuzumab (Herceptin^®^) for human epidermal growth factor receptor-2 (HER-2) overexpressing breast cancer was approved in 1998, revolutionizing treatment for breast cancer patients [16]. Additionally, imatinib demonstrated exceptional efficacy in chronic myeloid leukemia, with 98% of patients remaining in remission after five years [17]. In parallel, personalized functional drug testing platforms using pediatric tumor-derived cultures are increasingly being integrated into clinical care to guide individualized treatment decisions for relapsed and refractory cases [18]. Other targeted therapies like rituximab, gefitinib, and erlotinib have also improved outcomes and reduced side effects across various malignancies [19,20].

The widespread adoption of such an approach in pediatrics has lagged behind its adult counterparts, even though targeted therapy with entrectinib revolutionized the care for children harboring *NTRK* fusion for infantile fibrosarcoma [21]. NTRK fusions are recognized as rare but potent oncogenic drivers that promote constitutive kinase signaling and uncontrolled cell proliferation, with great clinical responses to inhibitors like entrectinib and Larotrectinib [22,23]. Similarly, ALK gene fusions play a critical role in the pathogenesis of pediatric anaplastic large cell lymphoma and neuroblastoma, providing highly actionable therapeutic targets [24,25]. To address these unmet needs, a multidisciplinary monthly molecular tumor board (MTB) was launched at Cook Children’s Medical Center (CCMC) in 2019 [26]. Initially, this consisted of monthly meetings with the physician teams to discuss patient cases and corresponding sequencing results. While this initiative was successful, the precision medicine team felt that its services were limited to a select few patients and needed to be applied more broadly. This led to the creation of a Precision Medicine Clinic (PMC), a revamped version of MTB, every week with tremendous growth of patient load and inclusion of multidisciplinary meetings reflecting change in standard practice. MTB does still exist independently every month and has changed focus to primarily educational topics.

Since its inception in 2021, the PMC at CCMC has evolved to fulfill the goal of precision medicine: providing comprehensive cancer treatment options based on genetic sequencing. The objective of this clinic is to provide comprehensive treatment options based on genetic sequencing to fulfill the overall goal of precision medicine in curing more patients and minimizing both short and long-term side effects associated with current cancer treatments. Our aims in initiating the precision medicine clinic were to provide universal next-generation sequencing (NGS) testing for pediatric cancer patients at CCMC, which would facilitate transitioning from the one-size-fits-all approach to treatment tailored for individual patients to more effectively avoid toxicity than traditional chemotherapy regimens. Hence, the purpose of this paper is to describe the experience of developing a precision medicine clinic at a large non-academic pediatric center and its impact.

## 2 Methods for Developing a Precision Medicine Clinic

### 2.1 Overview/Development

The development of our PMC was preceded by the creation of the MTB at CCMC. Since its inception in 2019, the MTB, comprised of a successful multi-disciplinary committee, facilitates the discussion of over 150 challenging cases, most of which had sustained an unfortunate relapse. The objective of the PMC was to create a centralized and standardized platform in which to universally compile, discuss, and provide recommendations on patient cases with NGS testing guided by the principles of precision medicine. This was achieved with the help of our patient registry, allowing us to track changes in tumor biomarkers and follow treatment decisions.

### 2.2 Workflow

**Figure 1:**
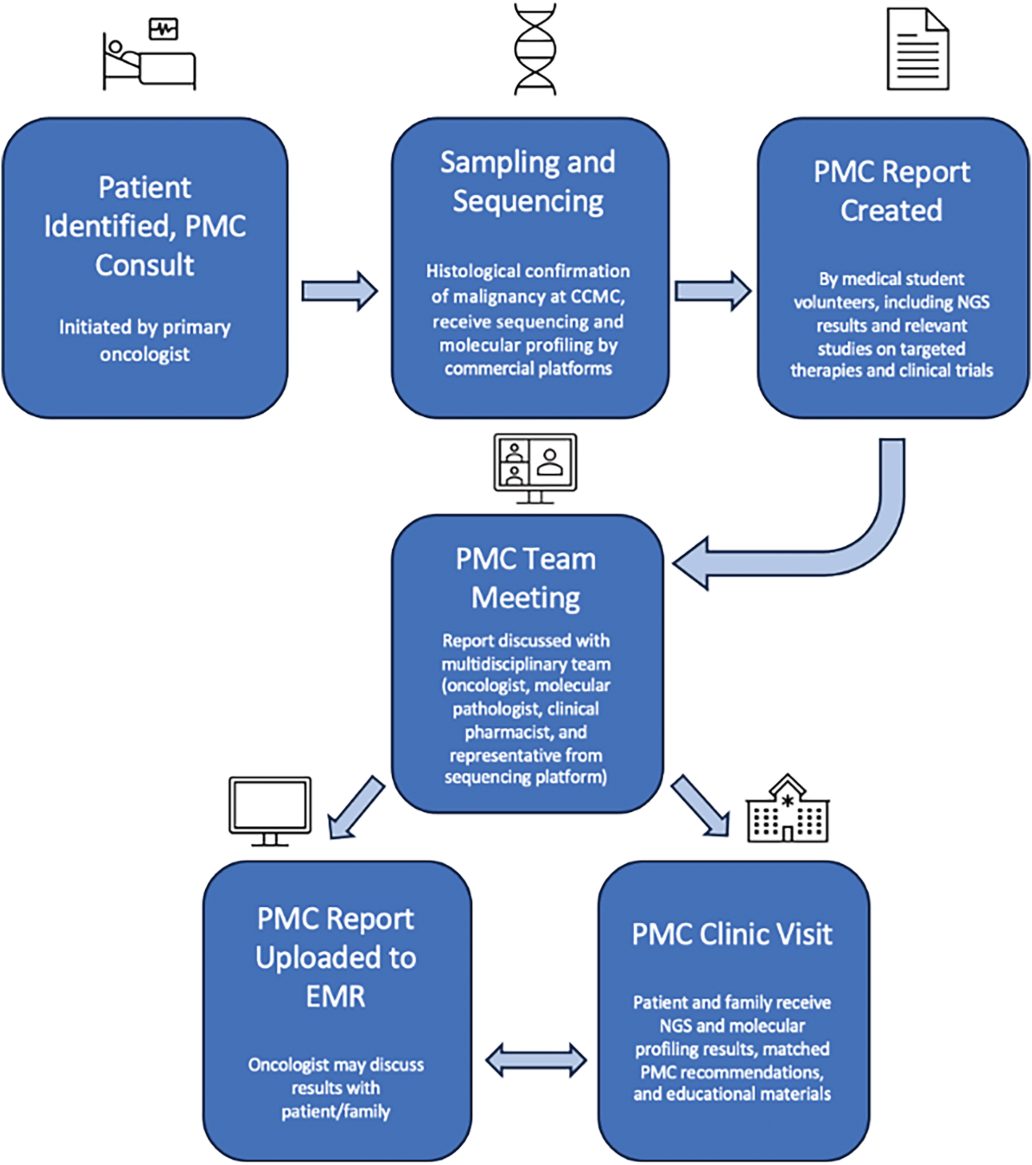
Precision medicine clinic (PMC) flow. CCMC, Cook Children’s Medical Center; NGS, Next Generation Sequencing; EMR, Electronic Medical Record. Figure created with Microsoft PowerPoint Software (Version 16.97 (25051114))

In many ways, the PMC duplicated the workflow and structure of the MTB with the addition of a dedicated clinic. This workflow, depicted in Fig. 1, begins when an eligible patient is identified. Tissue samples from the patient are sent to CCMC’s Pathology Department for histological analysis and to a commercial platform for sequencing and molecular profiling. Foundation Medicine Inc. (Cambridge, MA, USA), Tempus (Chicago, IL, USA), Caris Life Sciences (Irving, TX, USA), and Natera (Austin, TX, USA) were the commercial platforms used for sequencing. We investigated the available literature provided by each platform on their websites, detailing their testing methods and success; this guided our decision in choosing them for our sequencing. The FoundationOne test from Foundation Medicine Inc. utilizes next-generation sequencing (NGS) *in vitro* assays to detect the 4 main classes of genomic alterations: base substitutions, insertions and deletions, copy number alterations, and rearrangements. Cook Children’s Health Care System has previously documented the success of this testing in detecting actionable oncogenic variants leading to targeted therapy in twelve patients [27]. Sequencing is universal and completed for all cases involved. Standard analysis of tumor cells is performed by the CCMC’s Pathology Department, which includes microscopic examination to assess cell morphology, tissue architecture, and abnormal changes, Fluorescence *in Situ* Hybridization (FISH), and Polymerase Chain Reaction (PCR). Additional analyses may depend on the tissue of origin. The preliminary findings from these histologic processes guide the molecular profiling. It is important to note the classification of variants that may come back on genetic sequencing. Pathogenic variants are ones known to cause disease, with strong scientific evidence showing that the variant disrupts normal biological function, thus leading to some disorder or cancer. Likely pathogenic variants are changes that are strongly suspected to cause disease based on available evidence. Over time, with more research, these may eventually be confirmed as pathogenic. Variants of Unknown Significance (VUS) are DNA changes where it’s unclear whether they are harmful or harmless. There is not enough evidence to classify them as either disease-causing or benign, but they are included in reports in case more data on them becomes available.

### 2.3 Patient Selection

Upon launching the MTB, patient selection was limited to relapsed patients. As time passed, however, it was quickly apparent that NGS testing had potential applications for a larger patient population, with some evidence that treatment-naive patients may benefit more from targeted therapies. Due to a greater adoption and understanding of changing tumor genetics secondary to treatment, we elected to sequence all patients. The Pediatric MATCH clinical trial, which looks into precision medicine for solid tumors, is an important trial that has helped us turn our attention towards patients beyond those who have relapsed [28]. With this realization, the PMC aimed to order NGS tests for all newly diagnosed and relapsed oncology patients, followed by a careful review of the results and available literature. Due to the nature of the clinic as a clinical service rather than a clinical trial, there were no strict inclusion and exclusion criteria for patients. As the clinic has grown and adapted, with the understanding that tumor genetics may change with exposure to treatment, the goal is universal genetic testing within the clinic.

The primary oncologist on a patient’s care team may consult the precision medicine service. In select cases, the PMC has assumed full care of the patient in addition to reviewing NGS results and providing recommendations. If a germline mutation is identified or a genetic syndrome is suspected, a referral to genetic counseling is initiated.

### 2.4 Precision Medicine Clinic Reports and Meetings

PMC meetings consist of a case presentation about a single patient, followed by discussion and questions. The report is frequently created and presented by a medical student volunteer [29]. This is a unique feature of our clinic as it encourages medical students to engage in research about cutting-edge therapies and clinical trials. The reports are checked for accuracy and completeness by a pediatric oncologist prior to presentation.

The students are assigned a patient that will be presented at the weekly meeting and instructed to create a report that includes: a concise patient history, clinical course overview, relevant lab findings, sequencing test results, and a summary of treatment options specific to the NGS results. The student discusses the case with the lead oncologist and then performs an independent review of the available literature and current standards of treatment to provide a treatment summary. This is done with the guidance and assistance of both an oncologist and a research intern who are part of the PMC. These individuals are available to provide their expertise and experience in the specific case, as well as answer any questions. Once the report is done, it is submitted to the lead oncologist of the PMC and the oncologist(s) who are part of the patient’s treatment team before presentation at the meeting. During this time, the physicians can read the report and confirm the accuracy and completeness of the information provided. Each meeting is attended by oncologists, researchers, pharmacists, molecular pathologists, the solid tumor coordinator, medical students, and a medical science liaison from the platform used for the particular test. The document is then edited to reflect any discussion or recommendations from the team. This presentation, in addition to the results of the NGS and patient education material, is uploaded into the electronic medical record (EMR) and made available to the patient and their family. A prototype report is available under supplemental materials. Using an interdisciplinary team for the PMC allows for real-time review of research protocols, clinical trials, potential investigational agents, and commercially available options.

### 2.5 Precision Medicine Clinic Appointments

Patients referred to our PMC typically will attend 1–2 clinic visits following the PMC meeting, at which point their NGS results and treatment options are discussed. At these clinic visits, our nurse educators and PMC oncologists review the patient’s results in detail, provide the report generated from the patient’s PMC meeting, and are available to answer questions from the patient or their family. Our solid tumor coordinator helps the family navigate pursuing investigational and commercially available treatment options, including clinical trials. Regardless of whether the team meets with the individual patient/family, a consultation note is placed on the electronic medical record for future reference.

### 2.6 Precision Medicine Clinic Patient Registry

Our in-house RedCap registry allows us to track patients seen and discussed at our PMC, which is especially important when it comes to relapsed or treatment-resistant patients. Our registry includes demographic information on the patient, in addition to their histopathological diagnosis, pharmacogenomic testing (PGX), tumor origin (TO) as determined by the NGS testing, programmed cell death ligand 1 (PD-L1) status, microsatellite stability (MS) level, tumor mutation burden (TMB), specific genomic findings, RNA expression (over/under), variants of unknown significance (VUS), and the NGS company used and other miscellaneous NGS findings. Circulating tumor DNA (ctDNA) findings are now being included as well to more fully define tumor characteristics and correlate imaging with relapse predictability. The patient’s status in terms of the PMC meeting date and appointment date is also noted. Treatment options discussed at the PMC meeting, traditional treatment options (typically chemotherapy), targeted treatment options, and the targeted treatment or immunotherapy initiated post-receipt of NGS results are all stored in a database.

### 2.7 Treatment

The PMC focuses on developing first, second, and further lines of treatment that can then be captured in our secure RedCap database for future therapy needs. The patient’s demographic data and treatment options, as well as information on their treatment course, are stored in this in-house registry of molecular testing to track changes in their tumor biomarkers and follow treatment decisions that have been made, including those based on commercial NGS results. This data can be acted upon as clinical needs arise. The treatment lines that the PMC focuses on are those that are provided by the National Cancer Institute PDQ, as well as the National Comprehensive Cancer Network, if applicable, based on tumor type. Additionally, any pertinent available literature focusing on the different components of the case (tumor type, genetic variant, clinical presentation) is reviewed and discussed with the patient, family, and medical team.

## 3 Results

**Figure 2:**
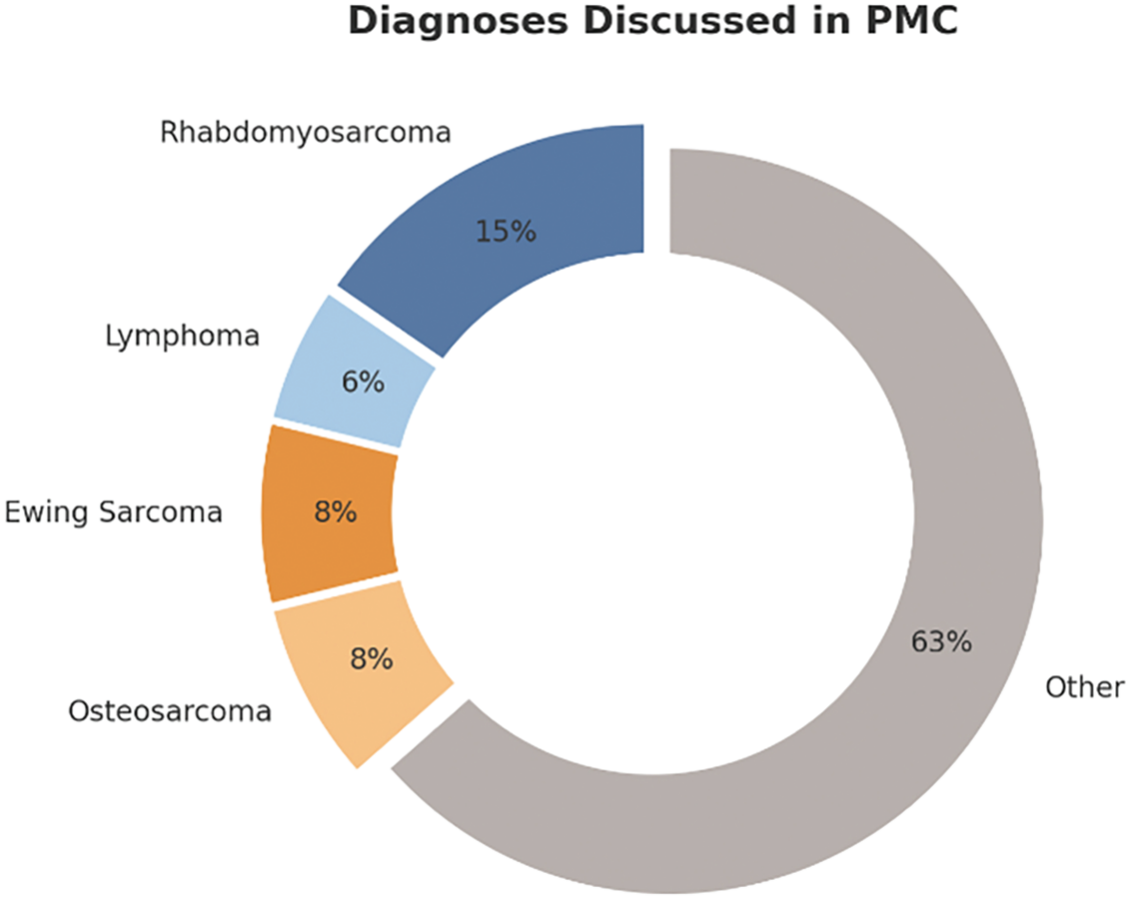
Breakdown of diagnoses discussed in the PMC for all 69 patients. Figure created with Microsoft PowerPoint Software (Version 16.97 (25051114))

The PMC began its virtual meetings in April 2022 and saw its first patient in the clinic in May 2022. 69 patients in total have been seen or discussed in PMC with a wide variety of diagnoses (Fig. 2) from 2022 to 2023. Before the creation of the PMC clinic, there was no panel to discuss these patients, nor a specific clinic to meet with them to discuss and explain their genetic sequencing results.

From patients discussed in our PMC, 133 genetic variants were found, with a total of 101 genes presented. These genes can be attributed to a multitude of oncogenic pathways, including, but not limited to, cell cycling, DNA processing and expression, and cell signaling (Tables 1 and 2).

**Table 1:** Incidence of genetic alterations by diagnosis, with genes grouped into pathways and PD-L1 status

Diagnosis	Pathway				PD-L1 status
	Cell cycling	RTK/GFRs	p53	Other	
Ewing sarcoma	7				
Rhabdomyosarcoma	4	1		3	
Osteosarcoma	2		2		
Myoepithelial carcinoma	1				+
Anaplastic large cell lymphoma		1			+
Diffuse large B-cell lymphoma		1			+
Inflammatory myofibroblastic tumor		1			+
Epithelioid sarcoma					+

**Table 2:** Genetic mutations by a pathway with the corresponding number of variants are seen in our PMC population

Cancer predisposition		Cell cycling		RAS/MAPK		RTK/GFRs		Other	
DICER1	1	CCNB3	1	BRAF	4	ALK	2	Total	19
TP53	4	CCNE1	1	KRAS	1	FGFR1	1		
		CDK4	2	MAP2K1	1	FGFR4	1		
		CDKN1B	1			KIT	1		
		CDKN2A	5			PDGFRB	1		
		CDKN2B	4						
		EWSR1	9						
		MSH3	1						
		MYC	1						
		MYCL1	1						
		RB1	1						

The patient’s immunotherapy markers are also recorded in the database and included as part of the report, including their PD-L1, microsatellite status, and TMB. Twenty patients were PD-L1 negative, while 14 patients stained positive for PD-L1 (>5% in any clone). This is further discussed in a separate paper [30].

Of the patients seen in PMC, four thus far have received recommended targeted therapy as a result of PMC involvement (Table 3). We are currently capturing outcomes data on the patients seen and discussed in our PMC and plan to disclose these results when sufficient data points become available to comment on.

**Table 3:** Patients with genetically actionable targets who received targeted therapy following the PMC meeting

Cancer diagnosis	Actionable target	Targeted therapies recommended by PMC	Targeted therapy initiated following PMC meeting
Colon Adenocarcinoma	BRCA1, PIK3CA, BRIP1, PIK3Ca BRCA1, TCF7L2 PPM1D APC FBXW7 CIC JAK1 FAT1 KRP1B NCOR1 FBXW7 PTEN LRP1B ASXL1 NOTCH3 PTCH1 FUBP1 TSC1 KMT2D SUZ12 APC KMTC(MILL3) JAK1 MSH6 DNM2 RAD50 GATA6 ATB BCL10 BCORL1 Germline PMS2	Dostarlimab, nivolumab + pembrolizumab, Nivolumab + Ipilimumab, Cetuximab or Panitumumab, Nipaparib, Olaparib, Recaparib, Talazoparib, Olaparib + Bevacizumab, Alpelisib	Nivolumab
Infantile fibrosarcoma	22P1		Larotrectinib
Osteosarcoma	KIT, PDGFRA, BCL2L2, KDR, RB1 G310fs*8 inversion exon 8, TP53 splice site, 993+1G>A	KIT, Sorafenib, Imatinib, Nilotinib, Sunitinib, 10 trials PDGFRA, Imatinib, 2 trials SARC038: Phase II Regorafenib + Nivolumab	Regorafenib, Pazopanib, Cabozantanib
Osteoblastic osteosarcoma	CCNE1		Regorafenib, Pazopanib, Cabozantanib

## 4 Discussion

### 4.1 Precision Medicine

Historically, the assumed “average patient” has been used as the model for which physicians make their recommendations to individuals. This one-size-fits-all approach was widely applied across specialties without the insight and data provided by a precision medicine approach. In fields like pediatric oncology, it is more appropriate to use precision medicine as it takes into account a patient’s genes, environment, and lifestyle [31]. Pediatric cancer patients have classically been enrolled in clinical trials or prescribed a standard treatment regimen based on their diagnosis, but these recommendations do not fully consider their precise genetic mutations. Potentially actionable results are found in 39%–95% of patients tested with a targeted panel, but only 5%–16% of these patients go on to be treated with approved or off-label drugs or clinical trials specific to these results [32].

Of the 69 patients reviewed by our precision medicine clinic, there were 133 unique genetic mutations, or an average of 1.93 mutations per patient. Thus, there is great variability in NGS results and vast amounts of data and literature must be sifted through and prioritized to come to a set of treatment recommendations as a result of genetic testing in pediatric oncology. Without the PMC, a thorough and standardized review of these results could not be so readily made available, and without such a review, the implementation of treatment recommendations based on NGS results would be unlikely. We propose that the PMC at our institution has paved the way for the use of the most up-to-date targeted treatments while expediting and simplifying the process of obtaining those treatment options. Utilizing a multidisciplinary team approach has provided the most efficient decision-making process based on NGS results, given the need for oncologists to triage large amounts of information and learn about new genetic variants with each patient. This necessitates the presence of other experts in genetics and pathologists to translate the clinical importance of the report results.

Direct comparison to other institutions with similar clinics is difficult due to differences in patient populations, protocols, and available resources. At our institution, we observe a gradual adoption of precision medicine, with the gradual implementation of recommended treatments. This reflects broader challenges in the field, where a lack of robust data and slow uptake of precision approaches remain common barriers across pediatric oncology initiatives.

### 4.2 Role of PMC and Clinical Trials

As previously discussed, the landscape of pediatric oncology differs from that of adults with pediatric cancer 5-year survival rate of 85% [33] compared to 68% for all adult cancers [34]. Many of the mutations found in pediatric cancers are not caused by environmental exposure but by gene fusions, which are potentially targetable for treatment [35,36]. These mutations may predict response to molecularly targeted therapies [37], but because of the relatively low frequency of recurrent genomic alterations in the same cancer and the low number of pediatric patients affected, studies specific to cancer type are challenging. Through genomic testing, the mutations that drive pediatric tumors have been identified regardless of tumor type, which may have unexpected similarities across diagnoses and age ranges. Thus, treatments used in adult oncology may be applied to pediatric oncology in some cases. By repurposing adult cancer drugs for pediatric tumors sharing the same genetic target, new clinical studies and potential treatments have been developed [3].

Not all DNA mutations are clinically actionable. DNA substitutions, fusions, deletions, and insertions are most likely to correlate with potential treatment options, depending on tumor type [38]. We found that 11 patients had gene fusions, the most common clinically-targetable type of DNA mutation. We present the mutations and associated pathways found in our Precision Medicine Clinic in this paper to share and illustrate the breadth and depth of information provided in uncharted territory for pediatric oncology with genetic testing that must be synthesized, highlighting the necessity of PMCs.

Additionally, PD-L1 testing is often a vital part of discussions about the care of this complicated patient population, who have typically undergone and failed multiple rounds of treatment. There is little in the literature about PD-L1 testing in this type of patient, who is the most common to undergo this type of testing at our large but non-academic institution [39]. These patients typically have been heavily pretreated with some combination of chemotherapy, surgery, and radiation, and subsequently have failed multiple traditional treatment modalities. PD-L1 expression testing is performed in these cases in the hope of providing salvage treatment options, often as part of clinical trials. Because PD-L1 testing is offered in many forms, when a patient is tested with all available techniques, the results yielded are significantly complex and delineate a variety of clones and expression measurements that must be correlated clinically for significance. If these results indicate in some manner that the malignancy may respond favorably to immunotherapy, then patients may be started on salvage treatments. The difficult reality of this method for pediatric oncologists is a complex and challenging road as they search for treatment options for refractory and rare malignancies. In sharing our experience with this method of testing, we hope to advance the exploration of the indications and implications of PD-L1 testing integration into precision medicine clinic discussions.

More and more clinical trials are being conducted in which patients are enrolled not by diagnosed cancer type, as has been the traditional guiding factor, but rather by coupling driving mutations and drugs targeting those mutations. This type of trial is known as a basket trial and is a new tool to study biomarker-targeted therapies for a variety of tumor types [40]. These trials take a patient who has the target feature and assign them to therapy and study the outcomes, rather than studying specific therapies for individual histologies. The goal is to determine which therapies show good response rates, but it can be difficult to interpret the results [40]. Currently, multiple Children’s Oncology Group (COG) trials are available at CCMC with this design, further exemplifying the increasing shift toward NGS as the standard of care for personalizing treatment for our pediatric patients. An example of such an approach is patients with an NTRK fusion being eligible to receive treatment with entrectinib. Our PMC is uniquely positioned to manage and interact with clinical trials of this design.

### 4.3 PMC and Survivorship

Another intriguing future direction for precision medicine in the context of PMC is its implication in cancer survivorship. After rounds of cytotoxic or irradiative therapies, most patients develop suboptimal and non-benign long-term side effects [41]. Survivor’s burden can include secondary malignancies and negative effects on the patient’s cardiac, endocrine, cognitive, and mental health, for which comprehensive and specialized care is required [42]. Targeted therapies identified based on NGS may help lessen these adverse effects and the survivors’ burden for cancer patients, and additionally reduce healthcare costs and utilization. All patients who are seen in the PMC are subsequently enrolled in the “Life After Cancer” program and are monitored regularly for organ health, late effects, and secondary malignancies.

Many clinical trials have demonstrated that targeted therapy is associated with superior outcomes and fewer drug toxicities, yet adoption of these therapy modalities has been slow to respond to this data and is not yet universal in pediatrics [26]. The decision to expand the utilization of NGS at our institution was in part due to this reluctance. The PMC is an attempt to normalize the practice for our physicians and create a streamlined process for genetic sequencing to be incorporated into the standard of care when determining treatment options for oncology patients.

### 4.4 Limitations

Limitations for our study, and more broadly, precision medicine, include that the current sequencing assays only test for a limited range of genetic mutations. Some known gene mutations are not included in the list of mutations tested for and are laboratory-dependent; therefore will not pick up on them, even if there is treatment for those mutations, we may be missing chances for targeted treatment. These technological shortcomings, paired with an overall hesitancy to implement novel therapies, have further limited the use of the PMC at our institution. Overall, we found that individual physicians were more inclined to conduct their discussion with the patient and family based on the PMC report presented at the meeting, as opposed to requesting a PMC appointment for the patient. Further development of technology, more data and studies, and more widespread utilization of these techniques are needed before the full potential of precision medicine and resultant clinics can be realized for pediatric cancer patients. Implementing precision medicine in pediatric oncology faces several challenges. Limited genomic data on childhood cancers, small patient populations, and the frequent identification of variants of unknown significance make it difficult to guide treatment [43]. Even when actionable mutations are found, targeted therapies may be inaccessible, unapproved for children, lack pediatric research on the specific medication, or be financially out of reach [44]. Ethical concerns and the psychological impact on families further complicate the integration of precision approaches into standard pediatric cancer care. Additionally, limitations to this specific description of the utilization of precision medicine include a single institutional description, small sample size, and lack of strict inclusion and exclusion of patients due to the continual nature of adaptation and growth of the PMC.

### 4.5 Future Directions

Due to the continual growth of the PMC at this institution, there are many additions currently in progress. First of all, standard operating procedures (SOPs) and standardization of data entry and management are at the forefront so that future analysis can be performed on the data from the clinic. A department of pharmacogenomics is currently being launched using genetic data to help inform and guide physicians on individualized patient metabolism and predicted side effect profiles. Additionally, a certification course for medical students involved in the PMC is being created. There is also currently research into how testing for circulating tumor DNA could impact the diagnosis and therapeutics for patients in the PMC. In the future, expansion of the PMC to non-cancer initiatives and other specialties may be of benefit to the hospital and patients.

## 5 Conclusions

The experience of creating a precision medicine clinic at Cook Children’s Medical Center has illuminated the great variety of uses for NGS in pediatric oncology as well as potential applications to other fields. Our experience has shown how a separate, dedicated task force is necessary to provide comprehensive services in interpreting genetic sequencing results, monitoring changes in patient status and broader scientific literature, and making well-informed and actionable targeted treatment recommendations in response to NGS results.

## Data Availability

The data that support the findings of this study are available from the corresponding author, LZ, upon reasonable request.

## Abbreviations

COG: Children’s Oncology Group
CCMC: Cook Children’s Medical Center
ctDNA: Circulating DNA
EMR: Electronic Medical Record
FISH: Fluorescence *in Situ* Hybridization
HER-2: Human epidermal growth factor receptor-2
PCR: Polymerase Chain Reaction
PD-L1: Programmed cell death ligand 1
PGX: Pharmacogenomic testing
PMC: Precision Medicine Clinic
MS: Microsatellite stability
MTB: Molecular Tumor Board
NGS: Next Generation Sequencing
TMB: Tumor mutation burden
TO: Tumor origin
VUS: Variants of unknown significance
